# Is fixation of both clavicle and scapula better than clavicle alone in surgical treatment of floating shoulder injury? A retrospective study

**DOI:** 10.1186/s12891-023-06583-8

**Published:** 2023-07-25

**Authors:** Yijie Shao, Xu Zhu, Bo Liu, Chenchen Ji, Jiajia Sun, Guangdong Chen

**Affiliations:** 1 Department of Orthopedics, The First Affiliated Hospital of Soochow University, 188 Shizi St, Suzhou, 215006 Jiangsu Province People’s Republic of China; 2 Department of Orthopaedics, the People’s Hospital of Leshan, 238 Baita Road, Leshan, 614000 Sichuan People’s Republic of China

**Keywords:** Floating shoulder injury, Surgical fixation, Glenopolar angle, Scapular fracture, Clavicle fracture

## Abstract

**Background & objective:**

Little research was available to explore which surgical fixation was better between fixation of both clavicle and scapula and clavicle alone in management of floating shoulder injury.

**Methods:**

Total 69 patients with floating shoulder injury receiving surgery from February 2005 to July 2020 participated in the study. 49 patients underwent fixation of the clavicle alone (Group C) while 20 patients underwent fixation of both clavicle and scapula (Group C + S). They were further divided into subgroups according to age: Group C1, Group C + S1 (age ≤ 55 years old) and Group C2, Group C + S2 (age>55 years old). The radiological parameter (glenopolar angle (GPA)) and clinical outcomes (Herscovici score, Constant-Murley shoulder outcome score (CSS score), and Visual Analogue Scale score (VAS score)) were collected and compared between these groups. The correlation between age and radiological parameter and clinical outcomes was calculated by the Spearman correlation analysis.

**Results:**

All people were followed up for at least 1 year. The degree of change in GPA before and after surgery in Group C + S is significantly better than that in Group C. The Herscovici and CSS score in Group C + S2 were significantly higher than those in Group C2 at 1 month, 3 months and 1 year after surgery. However, no significant difference in Herscovici and CSS score was found at final follow-up (1 year after surgery) between Group C + S1 and Group C1. The VAS score in Group C + S2 at final follow-up was significantly lower than that in Group C2. No significant difference in VAS score at final follow-up was found between Group C + S1 and Group C1. In addition, the VAS score was negatively correlated with Herscovici and CSS score. No correlation was found between VAS score and GPA.

**Conclusions:**

Both types of surgical fixation are effective in management of floating shoulder injury. For young people with floating shoulder injury, both types of surgical fixation are equally effective. However, for older people with floating shoulder injury, fixation of both clavicle and scapula is better in prognosis than fixation of clavicle alone.

## Background

The “floating shoulder injury” is a rare condition characterized by double disruptions of the superior suspensory shoulder complex (SSSC) resulting in both fractures of midshaft clavicle and ipsilateral scapula [1]. It usually results from high energy trauma with an incidence of approximately 0.10% of all trauma cases [2]. The floating shoulder can disrupt anatomic stability and may result in serious complications such as delayed union, malunion and nonunion [3]. In addition, it also can lead to subacromial impingement, muscle weakness, shoulder dysfunction and so on [4].

However, the treatment of floating shoulder injuries has been debated for many years and there is no consensus on the optimal treatment currently [5–7]. Kimia et al. believed that conservative treatment was recommended with nondisplaced or minimally displaced fractures while surgical treatment was appropriate for significantly displaced fractures especially scapular neck fractures [5]. Yadav et al. reported that the mean Herscovici score in operative group was significantly better than that in conservative group. They believed that the operation could improve GPA and give better functional outcomes when compared with conservative treatment [8]. However, no significant differences in clinical curative effect were found among conservative treatment, fixation of clavicle alone and fixation of both clavicle and scapula [7].

Many factors including fracture displacement, surgeon’s preference, patient’s age, patient activity requirements, degree of rehabilitation exercise and so on have a potential influence on treatment strategies for floating shoulder injury. The purpose of this study is to explore which surgical fixation was better between fixation of both clavicle and scapula and clavicle alone in management of floating shoulder injury.

## Methods

### Participants

This study is a retrospective study which enrolled in 69 patients with floating shoulder injury from February 2005 to July 2020. This retrospective study was approved by the Ethics Committee and Institutional Review Board of the First Affiliated Hospital of Soochow University (2022 − 413). 49 patients underwent fixation of the clavicle alone (Group C) while 20 patients underwent fixation of both clavicle and scapula (Group C + S). They were further divided into subgroups according to age: Group C1, Group C + S1 (age ≤ 55 years old) and Group C2, Group C + S2 (age>55 years old). Inclusion criteria were both fractures of midshaft clavicle and ipsilateral scapula meeting the definition of a floating shoulder and age between 20 and 75 years old. The exclusion criteria were as followed: (1) with neurovascular injuries; (2) open fractures; (3) previous history of fractures around the shoulder; (4) bilateral floating shoulder injury; (5) with rib fracture surgical intervention; (6) with ipsilateral upper limb fracture. (7) with pathologies such as cuff rupture-frozen shoulder. Internal fixation system for all patients was provided by Johnson & Johnson. We performed the fixation of scapular neck fractures via traditional Judet approach using 2.7-mm AO reconstruction plate [9]. In addition, we performed the fixation of clavicle fractures using 3.5-mm AO locking compression plate (LCP).

### Clinical and radiological parameters measurement

**Fig. 1:**
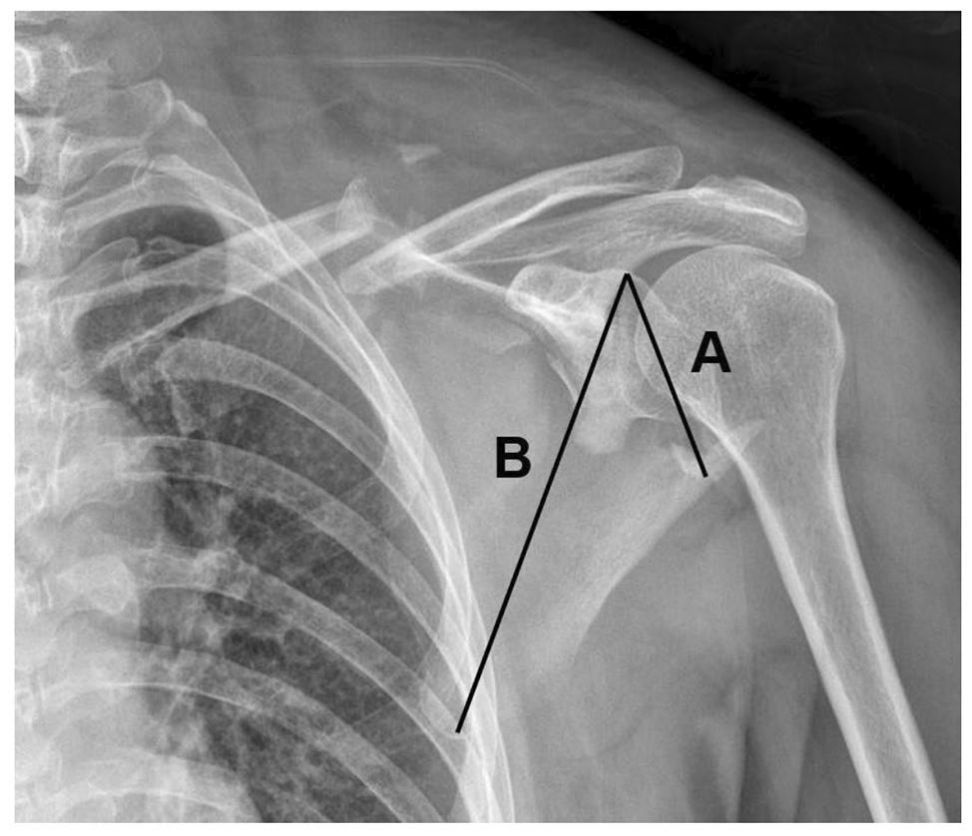
Measurement of the GPA

The clinical outcome was evaluated by the Constant-Murley shoulder outcome scoring system (CSS), the Herscovici scoring system and Visual Analogue Scale score (VAS) based on previous studies [1, 10]. The glenopolar angle (GPA) was used to reflect the radiological changes and was correlated with prognosis [8]. As shown in Fig. 1, the GPA was measured by the angle between line A (connecting the upper margin and the lower margin of the glenoid cavity) and line B (connecting the upper margin of the glenoid cavity and the lower margin of the scapular body) [11]. The GPA was measured in an anteroposterior axis perpendicular to the scapular plane by three different physicians independently.

The GPA was measured by the angle between line A and B. A is a line connecting the upper and lower margin of the glenoid cavity. B is a line connecting the upper margin of the glenoid cavity and the lower margin of the scapular body.

### Statistical analyses

All analyses were performed with Sigmaplot 14.0 (SystatSoftware, Inc). All data were described as mean ± standard deviation. The differences between different groups were analyzed with ANOVA tests. Pairwise comparisons were made with post-hoc corrections. The differences of Herscovici, CSS and VAS score during follow-ups among different groups were analyzed with repeated measurements of ANOVA tests. The correlation between age and radiological parameter and clinical outcomes were calculated by the Spearman correlation analysis. P value < 0.05 was considered statistically significant.

## Results

As shown in Tables 1 and 49 patients received fixation of the clavicle alone (Group C) and 20 patients received fixation of both clavicle and scapula (Group C + S) among 69 patients. Most patients were injured as a result of traffic accident and tumble. 55 patients had concomitant injuries such as rib fractures, head injuries, spinal fractures, lower limb fractures and so on. The operation time (P < 0.001) and length of stay (P < 0.05) in Group C + S were significantly higher than those in Group C. No significant differences were found between the young group and the old group for operation time and length of stay (Group C1 vs. Group C2, Group C + S1 vs. Group C + S2) (P > 0.05).

**Table 1:** Patient characteristics

Variables	Group C		Group C + S	
	Group C1	Group C2	Group C + S1	Group C + S2
Number	24	25	9	11
Gender (F/M)	4/20	3/22	1/8	3/8
Age (years)	42.4 ± 8.1	63.1 ± 4.5	43.4 ± 7.9	61.5 ± 4.0
Injury mechanism				
Traffic accident	7	5	2	5
Fall	0	2	1	1
Tumble	17	18	5	5
Crush	0	0	1	0
Injured limb				
Right	13	15	4	4
Left	11	10	5	7
Concomitant Injuries (with/without)	17/7	22/3	6/3	10/1
Operation time (min)	68.3 ± 22.2	60.6 ± 23.2	143.0 ± 46.7	155.4 ± 31.1
Length of stay (days)	9.3 ± 4.5	9.6 ± 5.6	15.4 ± 6.8	12.5 ± 6.5

Values are means ± SD

**Fig. 2:**
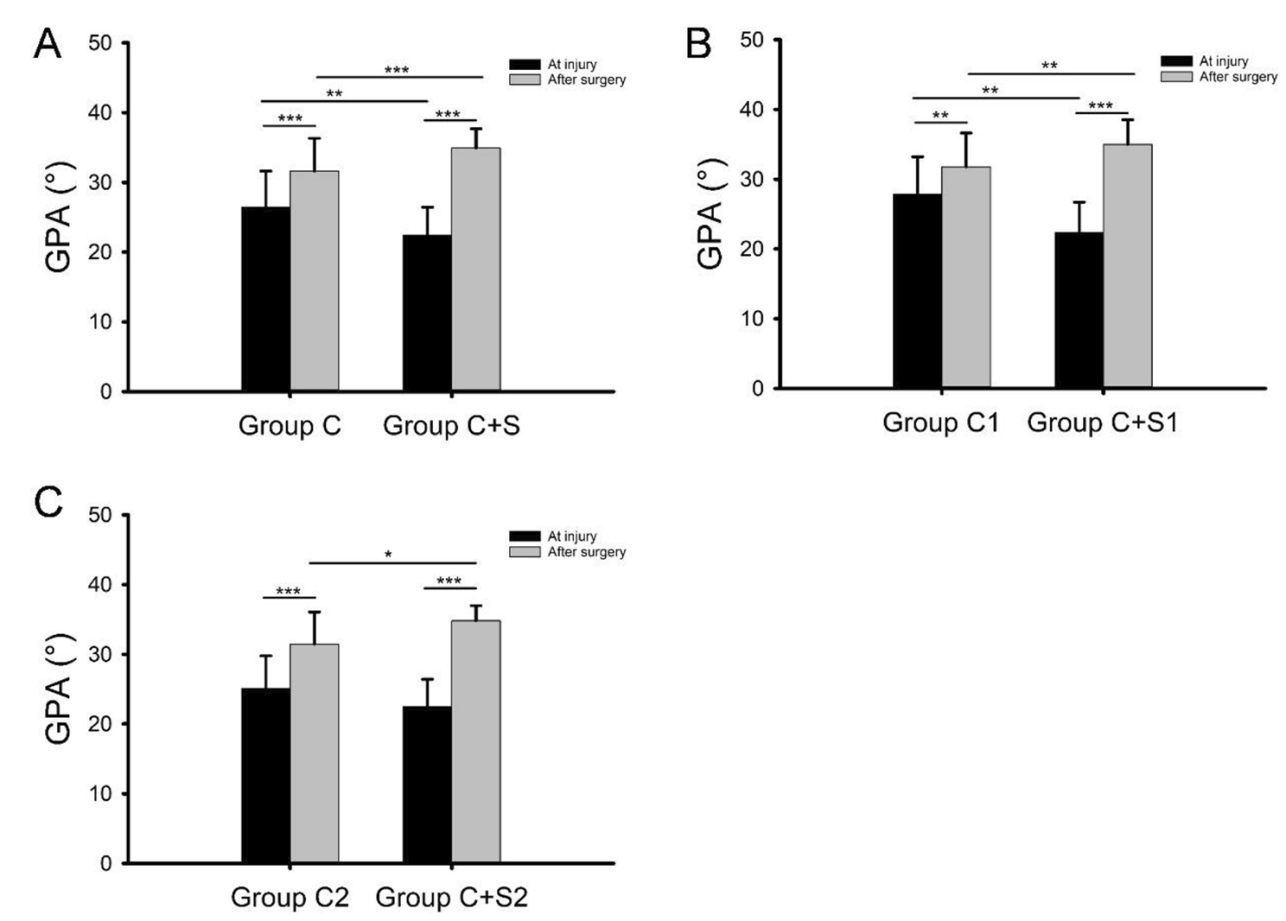
Comparison of GPA at injury and after surgery in each group and subgroups

The glenopolar angle (GPA) of patients in four groups before and after surgery were shown in detail in Table 2. The GPA at injury (P < 0.01) and after surgery (P < 0.001) in the Group C + S were significantly larger than that in the Group C (Fig. 2A). In addition, there were significant differences between GPA at injury and GPA after surgery in Group C and Group C + S (P < 0.001). The comparison of GPA in the subgroup (Group C1 and Group C + S1) was the same as that in the Group C and Group C + S (Fig. 2B). As shown in Fig. 2C, no significant difference was found in GPA at injury between Group C2 and Group C + S2 (P > 0.05).

**Table 2:** The radiological parameter (GPA) of patients in four groups

Variables	Group C		Group C + S	
	Group C1	Group C2	Group C + S1	Group C + S2
GPA at injury	27.9 ± 5.2	25.1 ± 4.6	22.3 ± 4.1	22.5 ± 3.7
GPA after surgery	31.8 ± 4.7	31.5 ± 4.5	35.0 ± 3.3	34.8 ± 2.1
Degree of change	3.9 ± 1.6	6.4 ± 3.3	12.7 ± 4.6	12.3 ± 5.1

Values are means ± SD

Comparison of GPA at injury and after surgery between Group C and Group C + S (A), Group C1 and Group C + S1 (B), Group C2 and Group C + S2 (C). *: P < 0.05; **: P < 0.01; ***: P < 0.001.

**Fig. 3:**
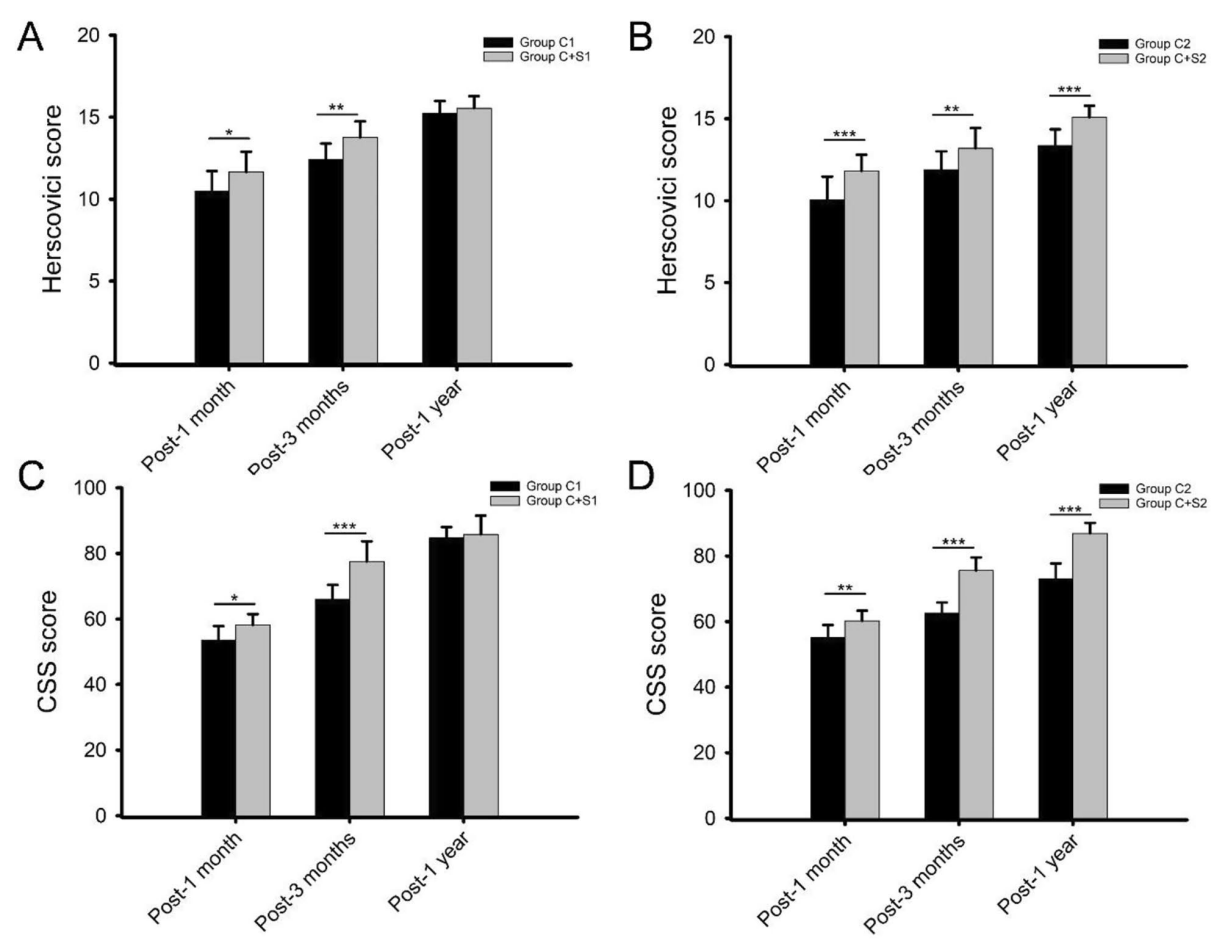
Comparison of Herscovici and CSS score at follow-ups in each group

The clinical outcomes (Herscovici score, CSS score and VAS score) of patients in four groups at follow-ups were shown in detail in Table 3. Figure 3 reflect the comparisons of Herscovici and CSS score at follow-ups in each group. There were no significant differences in Herscovici score, CSS score and VAS score at injury between groups. The Herscovici and CSS score in four groups increased significantly during postoperative follow-up (P < 0.05). In addition, the Herscovici and CSS score at 1 and 3 months postoperatively in Group C + S1 were significantly higher than those in Group C1 (P < 0.05). However, there were no significant differences in Herscovici and CSS score at 1 year postoperatively between Group C1 and Group C + S1 (P > 0.05) (Fig. 3A&C). Differently, significant differences in Herscovici and CSS score were found between Group C2 and Group C + S2 at 1 month, 3 months and 1 year postoperatively (P < 0.05) (Fig. 3B&D).

**Table 3:** The clinical outcomes of patients in four groups

Variables	Group C		Group C + S	
	Group C1	Group C2	Group C + S1	Group C + S2
Herscovici score				
Post-1 month	10.5 ± 1.2	10.0 ± 1.4	11.7 ± 1.2	11.8 ± 0.9
Post-3 months	12.4 ± 1.0	11.9 ± 1.1	13.8 ± 0.9	13.2 ± 1.2
Post-1 year	15.3 ± 0.7	13.3 ± 1.0	15.6 ± 0.7	15.1 ± 0.7
CSS score				
Post-1 month	53.6 ± 4.1	55.1 ± 3.8	58.2 ± 3.1	60.3 ± 3.0
Post-3 months	66.0 ± 4.3	62.6 ± 3.2	77.6 ± 5.8	75.5 ± 3.8
Post-1 year	84.7 ± 3.2	73.0 ± 4.6	85.8 ± 5.4	86.9 ± 3.1
VAS				
Post-1 month	5.9 ± 0.9	6.1 ± 1.0	6.2 ± 0.9	5.8 ± 1.3
Post-3 months	3.9 ± 0.8	4.5 ± 0.6	4.1 ± 0.9	3.9 ± 1.1
Post-1 year	1.2 ± 0.8	3.1 ± 0.7	1.7 ± 0.9	1.5 ± 0.8

Values are means ± SD

Comparison of Herscovici score at follow-ups between Group C1 and Group C + S1 (A), Group C2 and Group C + S2 (B). Comparison of CSS score at follow-ups between Group C1 and Group C + S1 (C), Group C2 and Group C + S2 (D). *: P < 0.05; **: P < 0.01; ***: P < 0.001.

**Fig. 4:**
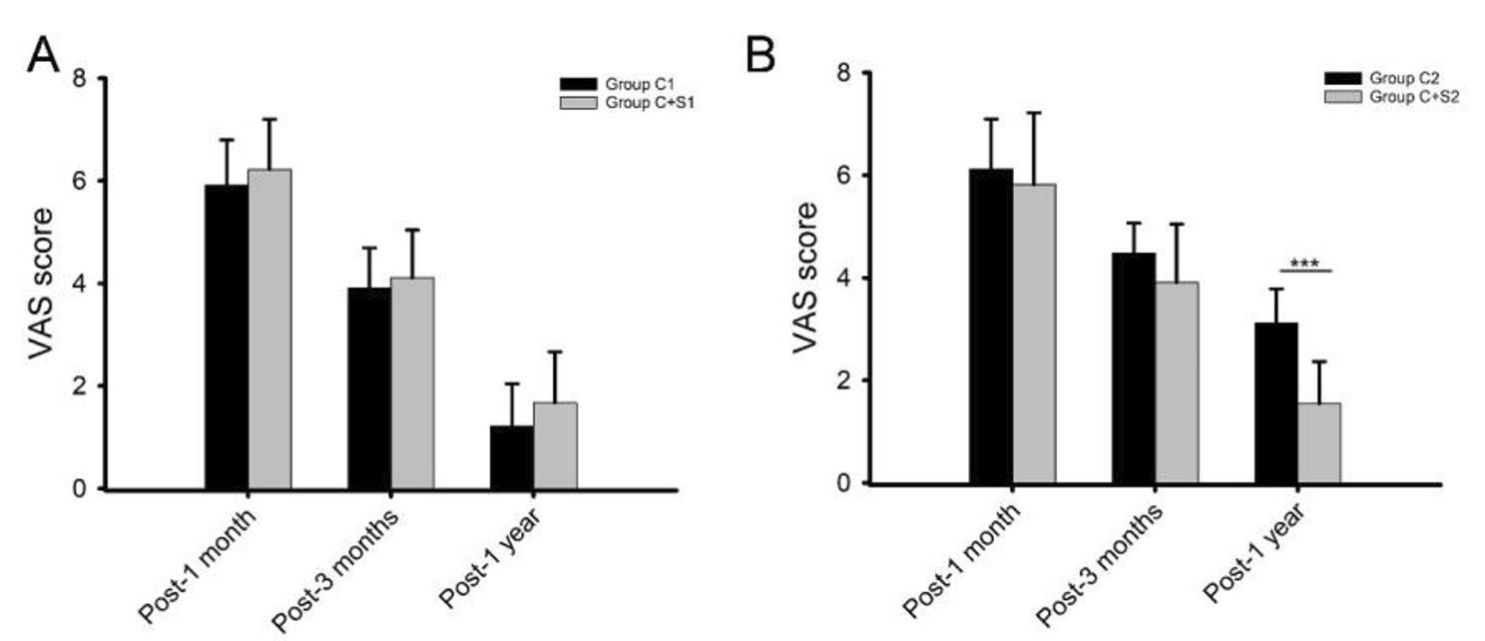
Comparison of VAS score at follow-ups in each group

As shown in Fig. 4, the VAS score in four groups decreased significantly during postoperative follow-up (P < 0.05). There was no significant difference in VAS score at each follow-up point between Group C1 and Group C + S1(P > 0.05) (Fig. 4A). On the contrary, the VAS score at post-1 year follow-up in Group C2 was significantly higher than that in Group C + S2 (P < 0.001) (Fig. 4B).

Comparison of VAS score at follow-ups between Group C1 and Group C + S1 (A), Group C2 and Group C + S2 (B). ***: P < 0.001.

**Fig. 5:**
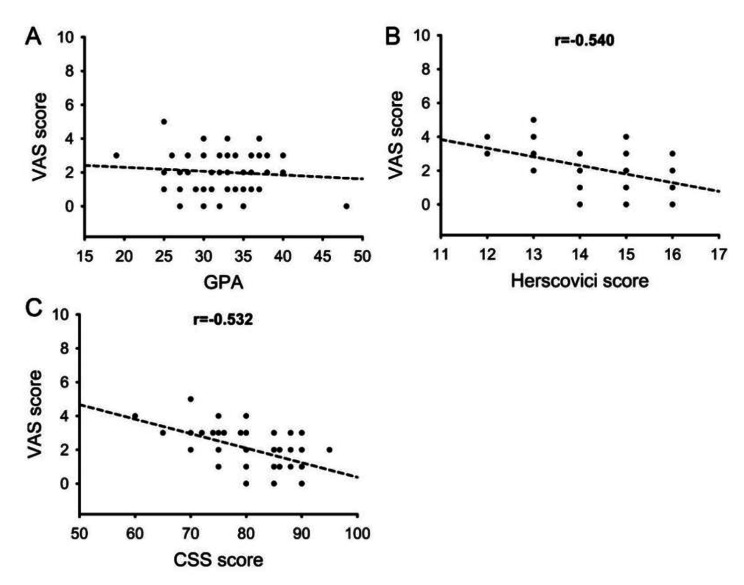
Correlation between radiological parameter (GPA) and clinical outcomes (Herscovici score, CSS score and VAS score) at 1-year follow-up

The correlation between radiological parameter and clinical outcomes at 1-year follow-up was performed in Fig. 5. The correlation coefficients between VAS score and Herscovici score, CSS score at 1-year follow-up was − 0.540 and − 0.532 respectively (P < 0.05). There was no significant correlation between GPA and VAS score (P > 0.05).

Correlation between VAS score and GPA (A), VAS score and Herscovici score (B), VAS score and CSS score (C) at 1-year follow-up.

**Fig. 6:**
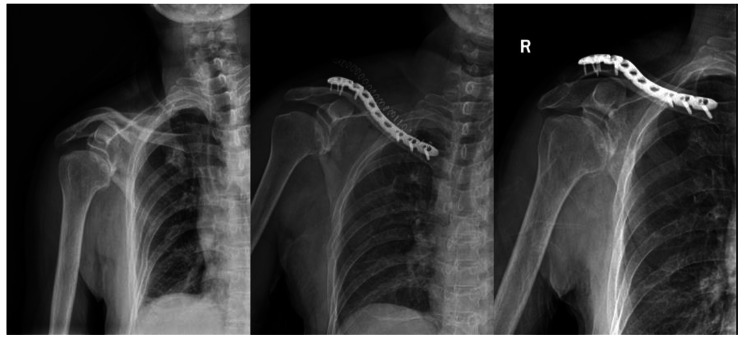
Case 1

**Fig. 7:**
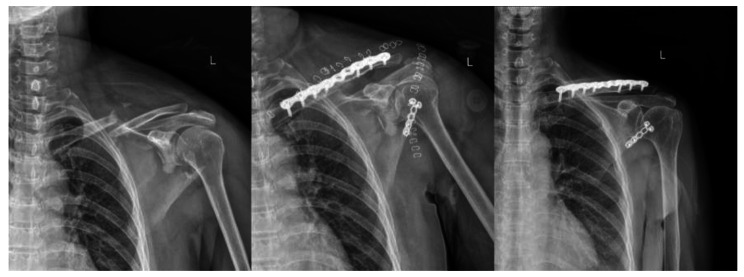
Case 2

Here are two cases suffered from floating shoulder injuries receiving different surgery fixations. Case 1 (Fig. 6) was a 63-years old man treated with fixation of clavicle alone. From the X-ray we could see that the fracture of scapular neck healed well 1 year after operation. Case 2 (Fig. 7) was a 57-years old woman treated with fixation of both clavicle and scapula. The displacement of scapular neck was apparent before surgery and was corrected well after surgery fixation. The fracture of clavicle and scapular neck healed well 1 year after operation.

A 63-years old man suffering from floating shoulder injuries was treated with fixation of clavicle alone. X-ray shows the pre-operation, 1 day after operation and 1 year after operation, respectively. The fracture of scapular neck healed well 1 year after operation.

A 57-years old woman suffering from floating shoulder injuries was treated with fixation of both clavicle and scapula. X-ray shows the pre-operation, 1 day after operation and 1 year after operation, respectively. The displacement of scapular neck was corrected well after operation. The fracture of clavicle and scapular neck healed well 1 year after operation.

## Discussion

Commonly, the floating shoulder injuries occur by high-energy trauma and accompanied by other complex injuries such as rib fracture, pneumothorax and so on. Many studies have compared the effectiveness of surgical fixation with conservative treatment for floating shoulder injuries and the results are still controversial [8, 12–14]. An increasing number of scholars are in favor of surgical fixation. Lin et al. enrolled 39 patients with the floating shoulder injuries and found surgical fixation of both the clavicle and scapula (n = 13) was superior to conservative treatment (n = 13) in GPA, CSS score and Disabilities of the Arm and Shoulder (DASH) score at follow-up [12]. Yadav et al. reported 25 patients with the floating shoulder injuries. 13 patients received conservative treatment and 12 patients received surgical treatment. They found that the mean Herscovici score in operative group was significantly better than that in conservative group. They believed that the operation could improve GPA and give better functional outcomes when compared with conservative treatment [8]. Jacopo et al. reported 122 adult patients with closed displaced midshaft clavicle fracture whom were treated with figure-of-eight bandage (F8-B). They found that after the application of the F8-B an residual displacement (RD) greater than 104% significantly increased the risk of delayed union. An RD greater than 140% was associated with a higher rate of nonunion [15]. In addition, Carlo et al. reported that 37 patients suffering from displaced midshaft clavicle fracture were treated nonoperatively with an F8-B. They found most of patients achieved good clinical results although 11 patients displayed delayed healing with an RD > 104% but less than 140%. They concluded that RD could have an impact on fracture healing and residual shortening (RS) could predict the functional clinical outcomes [15]. Our study found both fixation of clavicle alone and the fixation of clavicle and scapula were satisfactory in improving GPA, Herscovici and CSS score and decreasing VAS score at follow-up.

However, as for surgical fixation, there is no consensus statement on which is the better one between fixation of both the clavicle and scapular and fixation of clavicle alone. Gilde et al. reported clinical outcomes for patients with fixation of clavicle only and found that majority of patients could return to work despite some residual pain [16]. Oh et al. reported that no significant difference in clinical outcome was found between patients receiving different fixation (clavicle only or both clavicle and scapular). They further found unsatisfactory results in patients with displacement of scapular neck fracture > 1 cm [14]. However, Lin et al. compared the clinical and radiographic outcome of conservative treatment, fixation of the clavicle alone and fixation of both the clavicle and the scapula for patients with floating shoulder injuries. They concluded that patients receiving fixation of both the clavicle and the scapula had the best outcome. Our study compared the prognosis of fixation of the clavicle alone and fixation of both the clavicle and the scapula for patients in different age. We found that there was no significant difference in prognosis between two different fixations in young patients. On the contrary, the elderly patients who received fixation of both the clavicle and the scapula performed better function at final follow-up, which was consistent with other studies [17, 18]. Young patients with floating shoulder have strong muscle groups to stable the displacement of scapular neck fracture to relieve pain symptoms, which is conducive to functional exercise. On the contrary, the scapular fracture in the elderly patients will be unstable because of weak muscle strength and functional disorders of the rotator cuff may occur. The current study points out age is also an important factor in surgical administration of floating shoulder injuries.

Many studies found that the GPA had been proposed as an indicator for the outcomes of floating shoulder injuries [11–13, 19, 20]. Lin et al. found that the GPA after consolidation was positively correlated with DASH and Constant scores [12]. In addition, Kim et al. also confirmed that the GPA before surgery was significantly correlated with Constant scores [13]. Morey et al. concluded that restoring the GPA was likely to contribute to good clinical outcomes [2]. However, Yadav et al. found the correlation between the change of GPA and the Herscovici score was not significant. They also reported that no significant correlation was found between preoperative GPA and clinical outcomes at final follow-up in patients suffering from floating shoulder injuries [8]. Similarly, our study found that both fixations could improve GPA, however no significant correlation between GPA after surgery and clinical outcomes was found. The different results may be that the GPA is an angle measured in two-dimensional plane and not truly reflect the angulation between scapular neck and scapular body. CT scan may be helpful to visualize the angulation of the affected shoulder. Therefore, the GPA measured by X-rays may be not an appropriate indicator to reflect the prognosis in patients with floating shoulder injuries.

There are some limitations to our study. First, the number of patients for each group is limited because of the rarity of this injury. Second, the follow-up time was relatively short (the last follow-up period, 12 months). Third, CT scans were not undertaken to assess the healing outcome due to increased costs. The GPA is an angle measured in two-dimensional plane (X-ray) and not truly reflect the angulation between scapular neck and scapular body. CT scan may be helpful to visualize the angulation of the affected shoulder. Lastly, the time span of this study was over 15 years and the methods, used implants, and preferences were changing during this period. .Meanwhile, the healing outcome of post-operative patients will be further evaluated.

## Conclusions

In conclusion, our study found that both types of surgical fixation are effective in management of floating shoulder injury. For young people with floating shoulder injury, both types of surgical fixation are equally effective. However, for older people with floating shoulder injury, fixation of both clavicle and scapula is better in prognosis than fixation of clavicle alone. Besides the displacement degree, age is also a significant basis for the choice of surgical methods.

## Data Availability

The datasets used and analysed during the current study are available from the corresponding author on reasonable request.

## Abbreviations

GPA: Glenopolar angle
VAS: Visual Analogue Scale
CSS: Constant-Murley shoulder outcome score
SSSC: Superior Suspensory Shoulder Complex
